# Cardiac metastases from primary myxoid liposarcoma of the thigh: a case report

**DOI:** 10.1186/s12957-020-02009-0

**Published:** 2020-08-27

**Authors:** Kunihiro Ikuta, Tomohisa Sakai, Hiroshi Koike, Tohru Okada, Shiro Imagama, Yoshihiro Nishida

**Affiliations:** 1 Department of Orthopaedic Surgery, Nagoya University Graduate School and School of Medicine, 65 Tsurumai, Showa, Nagoya, 466-8550 Japan; 2 Medical Genomics Center, Nagoya University Hospital, 65 Tsurumai, Showa, Nagoya, 466-8550 Japan; 3 Department of Radiology, Japanese Red Cross Nagoya Daiichi Hospital, 3-35, Michishita, Nakamura, Nagoya, 453-8511 Japan; 4 Department of Rehabilitation, Nagoya University Hospital, 65 Tsurumai, Showa, Nagoya, 466-8550 Japan

**Keywords:** Cardiac metastasis, Myxoid liposarcoma, Extrapulmonary metastasis

## Abstract

**Background:**

Myxoid liposarcoma is well known to have an unusual proclivity for extrapulmonary metastasis. However, cardiac metastasis of myxoid liposarcoma is very rare, even in patients with advanced disease.

**Case presentation:**

A 40-year-old man was diagnosed with myxoid liposarcoma of the right thigh and treated with wide resection. Two years after the surgery, a low-density area in the left ventricle was found on follow-up chest computed tomography, and was suspected of being metastatic disease. He underwent surgical treatment, and the lesion was pathologically confirmed as metastasis of myxoid liposarcoma. Fifteen months later, he complained of slight dyspnea and developed metastatic disease in the right atrium. He was treated with surgical excision, followed by radiotherapy. Although there was no recurrence in the heart since the second cardiac metastasectomy, multiple metastases occurred in the abdominal cavity, lungs, and muscles. He finally died of the disease 2 years after the second cardiac metastasectomy.

**Conclusion:**

We experienced a case of primary myxoid liposarcoma in the thigh, accompanied by ectopic and metachronous cardiac metastases. Although this condition is rare, we should follow-up patients with myxoid liposarcoma, considering the possibility of cardiac metastasis.

## Background

Myxoid liposarcoma (MLS) is the second most common subtype of liposarcoma arising in children, adolescents, and young adults [1]. MLS usually occurs in deep tissues of the extremity, especially in the thigh. Some tumors have round cell areas that represent histologic progression to high-grade tumors. Round cells, defined as greater than 5% of the total cells, are associated with higher malignancy and metastatic potential, resulting in an unfavorable outcome in patients with MLS [2, 3].

MLS is known as a translocation-related sarcoma and has a translocation with FUS-DDIT3 or rarely EWSR1-DDIT3 fusion. Since the fusion protein resulting from these fusion genes acts as activated and deregulated transcriptional factors, it stimulates the proliferation of tumor cells [4]. Approximately one third of patients with MLS develop distant metastatic spread [5, 6]. Patients with MLS tend to have metastases to extrapulmonary sites, such as retroperitoneum, abdominal wall, abdominal cavity, and bone, even in the absence of pulmonary metastases [2, 7]. Although several authors have reported a high proportion of extrapulmonary metastases of MLS, ranging from 50 to 78% of all metastases [7–10], cardiac metastasis is extremely rare. Here we present a case of cardiac metastasis that occurred in a patient with primary MLS of the thigh.

## Case presentation

**Fig. 1:**
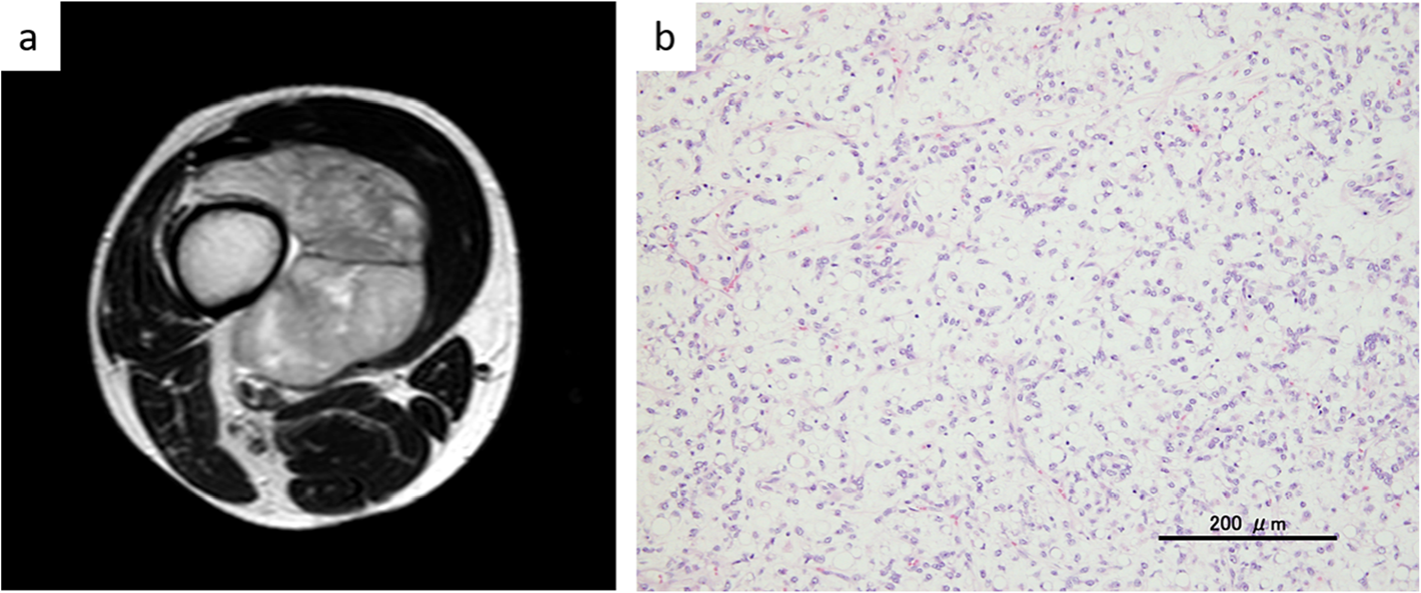
The findings at the initial diagnosis. **a** Axial T2-weighted MRI of primary myxoid liposarcoma of the right thigh showed a hyperintense mass adjacent to the distal femur. **b** The histological findings of the specimen in the resected primary tumor of the right thigh (hematoxylin and eosin, original magnification, ×200). A mixture of uniform oval non-lipogenic cells and small signet ring lipoblasts in a prominent myxoid stroma were observed

A 40-year-old man with a history of MLS in the right thigh was treated with wide excision at our hospital (Fig. 1a). At the time of diagnosis, he had no distant metastasis. Histological findings of the resected specimen revealed a round cell component of 10% and a negative margin (Fig. 1b). Adjuvant chemotherapy with four cycles of doxorubicin (70 mg/m^2^, every 3 weeks) was administered.

**Fig. 2:**
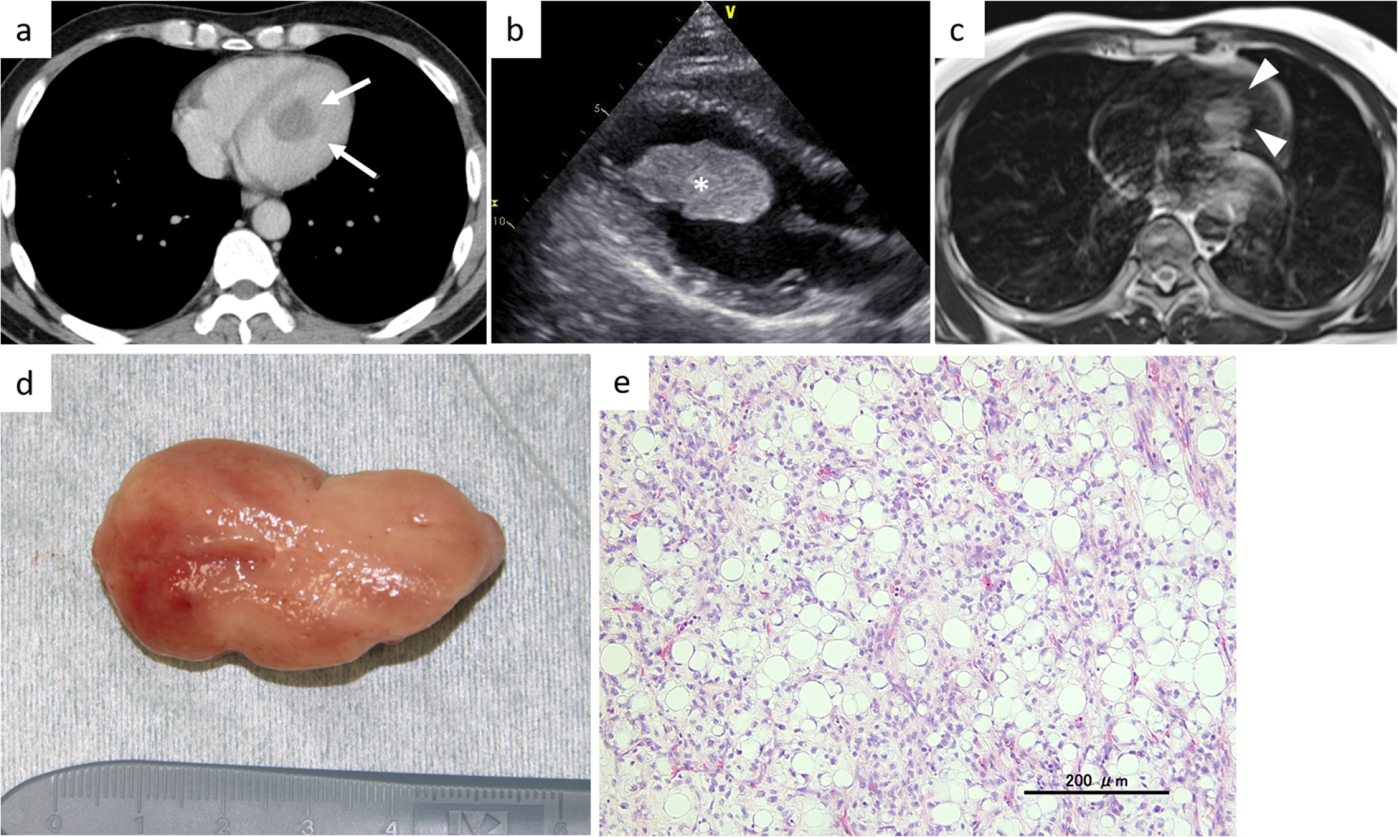
The findings at the first cardiac metastasis. **a** Chest CT scan showed a low-density lesion in the left ventricle (arrows). **b** Transthoracic echocardiography depicted a left ventricular mass, measuring 4 cm × 2 cm (asterisk). **c** Axial T2-weighted MRI demonstrated a high-signal intensity area compared to skeletal muscle within the mass (arrowheads). **d** Surgical material of the left ventricular tumor. **e** Photomicrograph of the resected specimen in the left ventricle showed increased areas of round cell component, compatible with metastasis of myxoid liposarcoma (hematoxylin and eosin, original magnification, × 200)

Two years after the surgery for the primary tumor, follow-up chest computed tomography (CT) showed a low-density area in the left ventricle. The patient was then asymptomatic. Contrast-enhanced CT showed a mass, measuring 4 cm × 2 cm in the left ventricle (Fig. 2a). Transthoracic echocardiography also identified the mass in the left ventricle, which was suggestive of a neoplasm (Fig. 2b). Clinical images revealed no evidence of local recurrence or distant metastasis other than the cardiac mass at that time. Given the risk of valve obstruction, he was immediately admitted to our hospital for cardiovascular surgery. On magnetic resonance imaging (MRI), the left ventricular tumor showed a lower-signal intensity than that of skeletal muscle on T1-weighted images, higher-signal intensity on T2-weighted images, and slight enhancement with a contrast agent (Fig. 2c). Considering the clinical course, the mass was regarded as a metastasis of MLS. He underwent surgical excision of the lesion in the left ventricle. Intraoperatively, we found a reddish-white tumor (Fig. 2d) arising from the papillary muscle without invasion of the interventricular septum. Histopathological examination of the specimen showed a mixture of oval non-lipogenic cells and small signet ring lipoblasts in a prominent myxoid stroma, which was consistent with the findings of the primary tumor in the thigh. However, the proportion of round cell component in the ventricular specimen was increased compared with that in the specimen of the primary tumor (Fig. 2e). A negative margin was histologically confirmed in the ventricular specimen.

Six months later, he complained of back pain and developed metastatic disease in the sixth thoracic vertebra. Conventional radiotherapy was administered to the spine lesion, which was delivered as 50 Gy in 25 fractions.

**Fig. 3:**
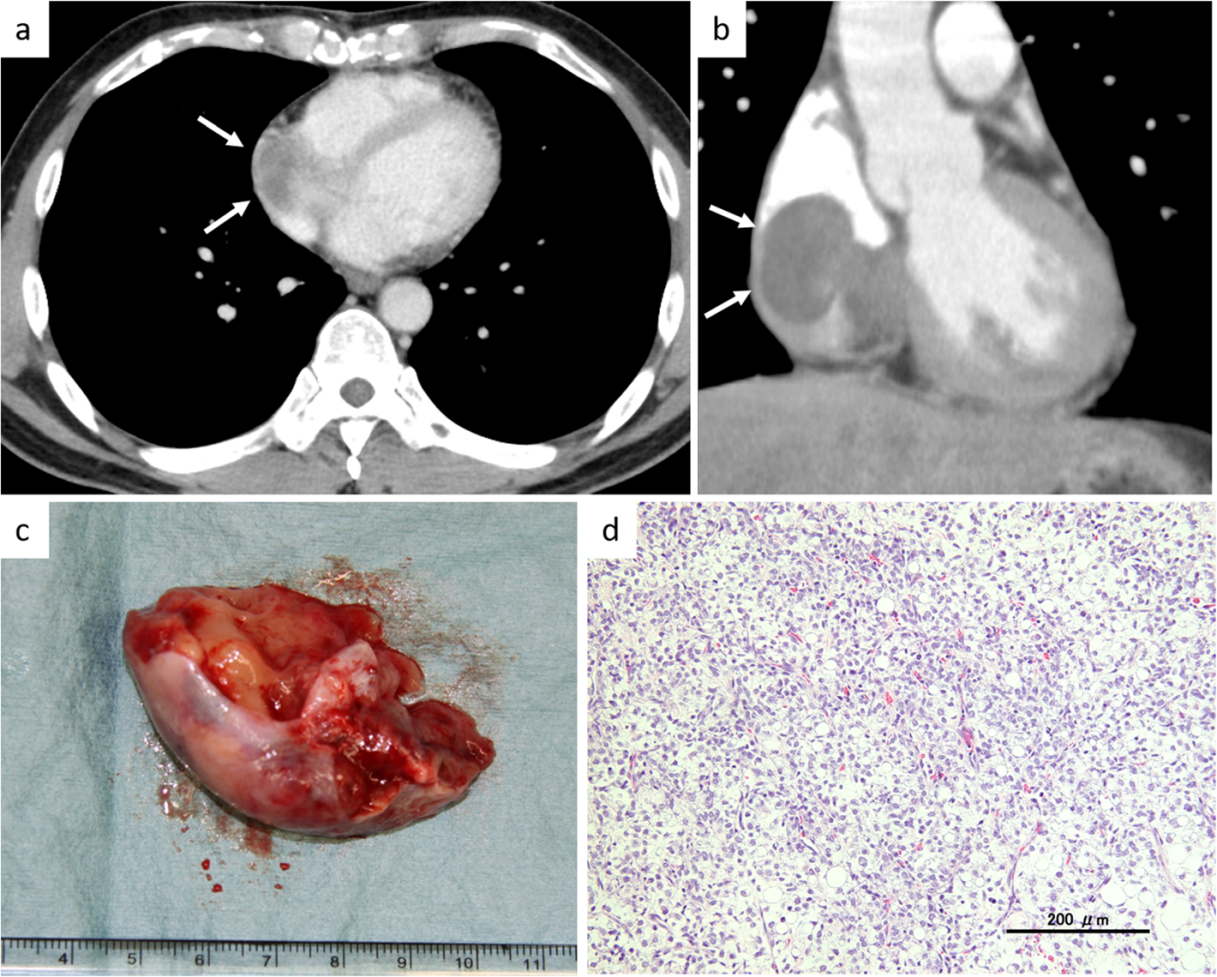
The findings at the second cardiac metastasis. **a** Axial and **b** coronal views of contrast-enhanced CT showed a metastatic tumor located in the right atrium (arrows). **c** The partially resected specimen from the atrial septum. **d** Photomicrograph of the resected specimen demonstrated hypercellular areas with round cell morphology (hematoxylin and eosin, original magnification, × 200)

Fifteen months after the cardiac metastasectomy, he presented slight dyspnea on effort. Contrast-enhanced CT showed a tumor in the right atrium involving the atrium septum (Fig. 3a). Our cardiovascular surgeons decided that the lesion was not amenable to complete excision with an adequate margin. The patient was treated with surgical excision of the tumor with R2 margin (macroscopically evident margin positivity) (Fig. 3b). The histological findings of the specimen indicated MLS with hypercellular lesions with a round cell component of about 30%, which represented progression compared to that of the left ventricular specimen at the first cardiac metastasectomy (Fig. 3c). Radiotherapy of 50 Gy in 25 fractions was performed for the residual disease of the right atrium and atrial septum postoperatively. His ejection fraction evaluated with echocardiography after the second cardiac metastasectomy remained 40%, and he could perform his daily activities without difficulty. There was no recurrence in the heart after the second cardiac metastasectomy, although multiple metastases occurred in the abdominal cavity, lungs, and muscles. Despite palliative chemotherapy with trabectedin and eribulin, he finally died of the disease 2 years after the second cardiac metastasectomy.

## Discussion and conclusions

Although cardiac metastasis from soft tissue sarcomas has been mentioned in the literature [11–19], early diagnosis of cardiac metastasis from soft tissue sarcomas is difficult for musculoskeletal oncologists because of its rarity. As to MLS, approximately thirty cases with metastasis to the heart, including pericardium, have been reported so far (Table 1). Most of these patients had disseminated disease. The time intervals between the onset of primary disease and cardiac metastasis were reported to be relatively long, ranging from 1 to 25 years [15–20]. The initial site of metastatic disease in our patient was the heart. He had a solitary cardiac tumor without obvious symptoms that presented 2 years after the primary surgery in the absence of metastases at other sites. Only 10% of patients with cardiac metastasis have been reported to show any symptoms [11]. This made it difficult to recognize the possibility of cardiac metastasis in our patient. On the other hand, he presented with slight dyspnea on exertion at the diagnosis of the second cardiac metastasis, probably because of inflow tract obstruction in the right atrium. Clinical symptoms depend on the location and extent of the lesion, which variously affect cardiac function. Careful physical examination alone is not sufficient for the early diagnosis of cardiac metastasis.

**Table 1:** Cases with cardiac metastasis of myxoid liposarcoma in the previous literature

Author	Year	Age	Gender	Symptom	Metastatic sites	Interval (years)
Tong et al.	1968	35	M	Dyspnea	LV	7
Godwin et al.	1981	59	M	Systolic murmur	RV, pericardium	25
Ravikumar et al.	1983	76	M	CHF	LV, pericardium	25
Lagrange et al.	1986	46	F	CHF	RV	7
Bartels et al.	1988	64	M	CHF	RV	3
Ozoux et al.	1988	60	M	Murmur	LV	17
Oshima et al.	1993	37	M	Not described	LV	5
Papa et al.	1994	45	M	Dyspnea	LV	15
Hatton et al.	1997	39	M	Angina	Pericardium	4.5
Sugiyama et al.	2000	61	F	CHF	RV	11
Ng et al.	2001	45	M	Arrhythmia	Interventricular septum	3
Lee et al.	2002	53	F	CHF	Pericardium	5
Wong et al.	2002	54	M	Dyspnea	RV	7
Fairman et al.	2004	56	F	Angina, syncope	LV	12
Kono et al.	2005	60	M	SVC syndrome	SVC, RA, RV	13
Aoyama et al.	2005	63	F	Dyspnea	Pericardium	1
Komoda et al.	2009	52	M	Dyspnea	RA, RV, arterio-ventricular sulcus	17
Lazopoulos et al.	2011	63	M	CHF	LV, pericardium, interventricular septum	13
Markovic et al.	2012	45	F	Angina	Pericardium	5
Fernández-Golfín et al.	2012	68	M	Not described	Pericardium	Not described
Pino et al.*	2013	-	-	-	-	-
Mottahedi et al.	2013	50	F	Dyspnea	RA, RV	4
Virtová et al.	2014	36	M	No symptoms	Interventricular septum	5
Xu et al.	2014	60	M	Dyspnea	RV	20
Farmer et al.	2014	61	M	CHF	LV	17
Motevalli et al.	2017	46	M	Dyspnea	LV, PA, pericardium	16

*M* male, *F* female, *CHF* congestive heart failure, *RA* right atrium, *RV* right ventricle, *LV* left ventricle, *PA* pulmonary artery, *SVC* superior vena cava

*Case series

The lung is the most carefully monitored organ for the development of metastases in soft tissue sarcomas. However, pulmonary metastases often occur with a time lag behind extrapulmonary metastases in patients with MLS. Estourgie et al. reported that 55% of MLS patients with metastatic disease had extrapulmonary metastases [7]. Nishida et al. noted that 50% of metastases occurred in extrapulmonary sites, even in MLS patients with round cell components of less than 5% [10]. The tendency to metastatic spread in extrapulmonary sites was attributed to an affinity for adipose tissues such as those in the retroperitoneum, subcutaneous tissues, and bone marrow [5]. If physicians depend on imaging of the chest and primary site as the initial staging and follow-up studies, a significant number of metastases may be missed due to the high incidence of extrapulmonary metastases in patients with MLS [21]. For the staging of MLS at diagnosis, the 2017 National Comprehensive Cancer Network (NCCN) guidelines recommended chest imaging and abdominal/pelvic CT as well as total spine MRI [22]. Recent studies suggest that whole-body MRI is the most reliable modality for surveillance of all likely sites of extrapulmonary metastases [6, 23]. Although our patient underwent chest CT and thigh MRI every 3–4 months and annual total spine MRI after surgery for the primary tumor, he did not received a follow-up with whole-body MRI.

Positron emission tomography is used as an alternative imaging modality to screen for metastases in patients with malignancies. However, metastatic lesions of MLS are likely to have low uptake on positron emission tomography [6]. False-negative results of positron emission tomography in patients with MLS have been widely documented, with a reported sensitivity as low as 14% for the detection of spinal metastases [21].

When a cardiac metastasis is found incidentally, it is usually incurable. In our patient, the disease in the right atrium was not a recurrence of the first cardiac metastasis in the left ventricle but was considered a metachronous metastatic lesion. Surgical treatment may not be the best option for patients with cardiac metastasis. However, in selected patients with no evidence of distant metastases, surgical excision of the cardiac metastasis, if technically feasible, provides a chance to prolong survival in life-threatening situations such as mechanical obstruction or valvular dysfunction [18, 19]. If the surgical margin is positive after cardiac metastasectomy, radiotherapy can be useful as a supplemental procedure since MLS is regarded to be radiosensitive. Although surgery is the mainstay of treatment for localized MLS, systemic therapy is often given to patients with locally advanced or metastatic MLS. Currently, doxorubicin, eribulin, and trabectedin are being used widely with some success in patients with advanced MLS.

To summarize, this report described a patient with MLS in the thigh, accompanied by ectopic and metachronous cardiac metastases. Periodic examinations with chest CT with careful attention may be able to document the presence of cardiac disease before the occurrence of severe cardiac complications. In patients with a solitary cardiac metastasis, especially when detected at an early stage, surgical excision, followed by some adjuvant therapies, can be the treatment of choice.

## Data Availability

All data generated or analyzed during this study are included in this published article.

## Abbreviations

MLS: Myxoid liposarcoma
CT: Computed tomography
MRI: Magnetic resonance imaging
